# Shedding of Syncytiotrophoblast-Derived Extracellular Vesicles Is Increased in Placenta Previa and Accreta Spectrum

**DOI:** 10.1007/s43032-024-01491-1

**Published:** 2024-03-07

**Authors:** Chiara Tersigni, Nicoletta Di Simone, Donatella Lucchetti, Filomena Colella, Marianna Onori, Silvia Perossini, Annalisa Vidiri, Rita Franco, Alessandro Sgambato, Manu Vatish, Antonio Lanzone, Giovanni Scambia, Anna Franca Cavaliere

**Affiliations:** 1 Dipartimento di Scienze della Salute della Donna e del Bambino e di Sanità Pubblica, Fondazione Policlinico Universitario A. Gemelli IRCCS, L.go A. Gemelli 8, 00168 Rome, Italy; 2 Istituto di Clinica Ostetrica e Ginecologica, Università Cattolica del Sacro Cuore, L.go A. Gemelli 8, 00168 Rome, Italy; 3 Department of Biomedical Sciences, Humanitas University, Via Rita Levi Montalcini 4, Pieve Emanuele, 20072 Milan, Italy; 4 IRCCS Humanitas Research Hospital, 20089 Rozzano, Milan Italy; 5 Dipartimento Universitario di Medicina e Chirurgia Traslazionale, Università Cattolica del Sacro Cuore, Rome, L.go F. Vito 1, 00168 Rome, Italy; 6 Nuffield Department of Women’s & Reproductive Health, University of Oxford, John Radcliffe Hospital, Oxford, OX3 9DU UK; 7 Department of Obstetrics and Gynecology, Gemelli Isola Hospital, Via di Ponte Quattro capi, 39, 00186 Rome, Italy

**Keywords:** Placenta previa, Placenta accreta spectrum, Syncytiotrophoblast extracellular vesicles, Biomarker, Invasion

## Abstract

Placenta accreta spectrum (PAS) refers to excessive placental invasion into the maternal uterus and it is associated with high risk of obstetric haemorrhage and adverse maternal-neonatal outcomes. Currently, no specific circulating biomarkers of PAS have been identified. Given that in PAS disorders, the depth and the extension of placental invasion into the uterus are expected to be increased, in this study, we analysed plasma levels of syncytiotrophoblast-derived extracellular vesicles (STBEVs) in women with placenta previa (PP), at a high risk of PAS disorders, and pregnant women with normal placentation. Venous blood samples were collected from 35 women with ultrasonographic diagnosis of PP and 35 women with normal placentation, matched for gestational age. Plasma samples were ultracentrifuged at 120.000 g to collect extracellular vesicles (EVs). To identify and quantify plasma placenta–derived EVs (or STBEVs), EVs were analysed by flow cytometry using a monoclonal antibody against placental alkaline phosphatase (PLAP). Plasma levels of STBEVs were significantly higher in PP patients compared to controls. Plasma levels of STBEVs in women with PP and PAS showed a trend to a higher concentration compared to women with PP without PAS, although not reaching a statistical significance. Circulating STBEVs are potential candidates as biological markers to be integrated to ultrasonography in the antenatal screening programme for PAS. More studies are needed to confirm our observation in a larger cohort of patients and to analyse a possible association between high circulating levels of STBEVs and PAS.

**Supplementary Information:**

The online version contains supplementary material available at 10.1007/s43032-024-01491-1.

## Introduction

Placenta accreta spectrum (PAS) is a pathological condition characterised by excessive placental invasion into the maternal uterus and high risk of obstetric ante- and per-partum haemorrhage [1–3]. Recently, the incidence of PAS has been growing because of the concomitant increase of its main risk factors such as caesarean section (CS) rates, advanced maternal age, and use of assisted reproductive techniques [4–7]. The current prevalence of PAS has been estimated to be about 1 in 400 cases in Europe [8] and up to 1 in 270 pregnancies in United States [4].

An additional main risk factor for PAS is placenta previa (PP), defined as the complete or partial covering by the placenta of the cervix’s internal uterine os. PP is most often diagnosed by ultrasound and strongly predisposes to PAS. This risk increases with increasing levels of surgery and is tied to placental implantation at the decidual/myometrial scar site (following uterine surgery, e.g. CS, myomectomy, curettage).

A recent British meta-analysis has estimated a prevalence of PP in all pregnancies of 0.56%, a prevalence of PP associated with PAS of 0.07%, and an incidence of PAS in women with a PP of 11% [9].

In clinical practice, the most challenging issue for obstetricians remains to antenatally diagnosis PAS and to anticipate and/or prevent peri-partum complications, improving maternal-neonatal outcomes.

In expert hands, ultrasound has high sensitivity and specificity [10, 11], although the diagnosis of PAS by ultrasonography remains highly operator dependent. On this basis, the aim of the current translational research is to identify circulating biomarkers of PAS to be integrated into ultrasound routine assessment for the implementation of a standardised prenatal diagnosis protocol.

Syncytiotrophoblast-derived extracellular vesicles (STBEVs), released from the placenta into maternal circulation throughout pregnancy, have great potential as a ‘liquid biopsy’ of the placenta [12]. STBEVs are detectable in the plasma of pregnant women from early first trimester and their levels increase progressively from first to third trimester of pregnancy [13]. Interrogation of STBEV reflects the health status of the syncytiotrophoblast in real-time and the STBEVs cargo is potentially a rich source of biomarkers of placental abnormalities.

Given that in PAS disorders, the depth and the extension of placental invasion into the uterus are expected to be increased, we hypothesised that the release of STBEVs into maternal blood might be higher in women with PAS disorders. The aim of this study was to analyse plasma levels of STBEV in a prospective cohort study involving women with PP, who showed the highest risk of PAS disorders, and pregnant women with normal placentation, at the same gestational age.

## Materials and Methods

### Study Population

This study was conducted at the outpatient clinics, wards, and delivery room of the Operating Unit of High Risk Pregnancy of the Fondazione Policlinico A. Gemelli IRCCS of Rome, Italy. The study was approved by the Ethics Committee of the Fondazione Policlinico A. Gemelli IRCCS of Rome (ID:4299 date of approval 23/01/2023) and conducted following the principles of the Declaration of Helsinki. Written informed consent was collected from all patients recruited in the study.

Pregnant women referred to our unit for diagnosis of PP in the period from January 2023 to June 2023 were recruited. The selection of patients diagnosed with PP was performed according to the ISUOG international guidelines [14]. The diagnosis and degree of PAS were defined by pathological assessment of paraffin-embedded inclusions of the placenta *in toto*, according to the International Federation of Obstetrics and Gynaecology (FIGO).

Women with uncomplicated pregnancy were recruited among those referred to our centre for regular outpatient obstetric check-ups. Exclusion criteria were maternal infectious diseases, autoimmune diseases, multiple pregnancies, pre-eclampsia, intrauterine growth restriction, type 1 and 2 diabetes, and congenital fetal abnormalities.

### Samples Collection

Three millilitres of venous blood were collected from each patient recruited in this study at diagnosis of PP by ultrasound, between 28 and 37 weeks of gestation, or in controls, matched for gestational age, via venipuncture from the antecubital fossa. Each sample was centrifuged at 1200 g for 10 min at 20 °C to remove the cellular component and obtain the plasma and was, then, frozen in 500 μl aliquots at − 80 °C until further use. All women recruited in this study delivered in our centre and data related to obstetric outcomes and pathological examination of the placenta were analysed.

### Isolation of STBEV from Plasma

Plasma samples were thawed at R/T. Each sample was diluted (1:1 vol/vol) with PBS, centrifuged at 3000 g for 30 min at 4 °C and, then, ultracentrifuged at 120,000 g for 90 min at 4 °C using an Optima XPN ultracentrifuge (Beckman Coulter). After ultracentrifugation, the supernatant was removed, and the pellet, containing extracellular vesicles of various sizes, was washed with PBS and centrifuged again at 120,000 g for 90 min at 4 °C. Finally, the supernatant was discarded, and the EV pellet was resuspended with 100 μl of PBS. To quantify STBEV, samples were first lysed with 0.25% NP40 for 60 min; the protein concentration was then determined by Bradford assay.

### Western Blot

For the analysis of PLAP expression, STBEV samples isolated from the plasma of patients with PP (*n* = 35) and patients with normal placentation (*n* = 35) were lysed on ice for 30 min with NP40 at 0.25% for 60 min. For each sample, a total of 20 μg protein was loaded. Protein content normalisation was performed by ponceau. Samples were denatured at 95° and then subjected to separation by SDS/PAGE (Invitrogen). Subsequently, semi-dry transfer was performed on a PVDF membrane (Biorad, Hercules, CA, USA). Non-specific bands were blocked with TBS-T (20 mM Tris/HCl, 137 mM NaCl, 0.1%Tween-20, pH 7.6) containing 5% milk (Santa Cruz Biotechnology Inc., Dallas, TX, USA) for 1 h at R/T. The membranes were incubated in saline buffer and 0.05% Tween 20 (TBS-T) containing 1% BSA at 4 °C overnight with a murine monoclonal anti-PLAP antibody (NDOG2, University of Oxford, Oxford, UK; 1 μg/ml), specific for syncytiotrophoblast, recognising placental alkaline phosphatase (PLAP), and a rabbit monoclonal anti-CD63 antibody (Abcam, UK), a marker of exosomes.

The membranes were washed in TBS-T before incubation with horseradish peroxidase–conjugated secondary antibody (1:4000; Dako, Glostrup, Denmark) for 1 h at R/T. The antibody used was diluted in the blocking buffer. After washing, the membranes were treated with a chemiluminescence system (PierceTM, Thermo Fischer Scientific, Waltham, MA, USA) and exposed to Hyperfilm ECL (GE Healthcare Life Sciences, Cleveland, OH, USA). Western blot densitometric analysis was made using Nine Alliance software (Uvitec Alliance, Cambridge, UK).

### Flow Cytometric Analysis of STBEVs

The analysis of STBEV was performed by multi-colour flow cytometry using a CytoFLEX S cytometer (Beckman Coulter) equipped with violet laser (405 nm) excitation sources. This instrument is capable of recording the SSC (side scatter) for the blue laser (BSSC) and the violet laser (VSSC). The flow cytometer was calibrated using the Megamix-Plus FSC beads, which emit FITC of different sizes (100, 300, 500, and 900 nm). The number of STBEVs was measured using the instrument’s cell counting function based on a calibrated peristaltic pump per loaded sample. STBEVs were marked with CellTraceTM Calcein Violet (ThermoFisher Scientific, Waltham, MA, USA) to distinguish intact vesicles from cell debris.

Before the STBEV samples were run, the filtered PBS was analysed three times for 2 min to remove the background (approximately 100 events per second). To confirm the placental origin of the vesicles, STBEVs were labelled with an anti-PLAP (placental anti-alkaline phosphatase) PE (phycoerythrin)–conjugated murine monoclonal antibody (NDOG2, University of Oxford, Oxford, UK) or its control IgG1 PE-conjugated (Biolegend UK Ltd., Cambridge, UK). PLAP protein is mainly expressed in placental tissue (https://www.proteinatlas.org/) and it is commonly used to distinguish vesicles released from the placenta from those derived from other cell types [15]. PLAP-positive vesicles were normalised to positive calcein events. EVs isolated from colorectal cancer cell culture medium were used as a negative control to verify PLAP positivity. Before use, all antibodies were filtered through Nanosep 0.2 mm centrifuge devices (Pall Life Sciences) to minimise interference from background particles. All antibodies were also titrated to ensure they were used at optimal concentrations. Fluorochrome compensation for the multi-coloured STBEV cytofluorometer was performed using BD Compbeads (BD Biosciences), labelled with fluorescence-conjugated antibodies.

All samples were incubated for 1 h at 4 °C in the dark with the anti-PLAP PE-conjugated antibody (NDOG2, University of Oxford, Oxford, UK) or its control IgG1 PE-conjugated (Biolegend UK Ltd., Cambridge, UK). Subsequently, filtered PBS was added to the labelled samples to reach 500 μl and evaluated immediately in the cytofluorometer (Beckman Coulter). The gates were initially organised so that ≤ 1% of the positive STBEVs fell within the negative controls. Data were acquired and analysed by CytExpert 2.2TM software (version 2.2, CytoFLEX S, Beckman Coulter, Milan, Italy).

### Statistical Analysis

The clinical data are shown as the mean ± standard deviation (SD) or percentage (%), depending on the type of variable, and were analysed using Student’s *T* test. All data was analysed for normal distribution using the Shapiro–Wilk test and analysed by the Mann–Whitney *U* test performed with Prism software version 9.0. For all analyses, *p* < 0.05 was considered significant.

## Results

### Characteristics of the Study Population and Obstetric Outcomes

In this study, we recruited 35 women with PP and 35 with uncomplicated singleton pregnancies, matched for gestational age (gestational week at venous blood sampling), for the analysis of circulating levels of STBEVs.

Clinical characteristics of the study population are summarised in Table 1. A significant difference between patients with PP and the control group was found in terms of age, use of assisted reproductive techniques (ART), and prior history of curettage, caesarean section, and endometriosis. Concerning the gestational outcomes, women with PP showed lower gestational age at delivery and neonatal birth weight and higher intrapartum estimated blood loss (EBL). The placentae from all women with PP were investigated for PAS by expert pathologists. Among the 35 PP cases, 6 patients had a confirmed diagnosis of PAS.

**Table 1:** Clinical characteristics and obstetric outcomes of women recruited in the study

	Placenta Previa (*n* = 35)	Controls (*n* = 35)	*P*
Age	35.8 ± 5.0	33.4 ± 4.4	**< 0.05**
Primiparous (*n*)	16 (46%)	18 (51%)	0.63
BMI	23.4 ± 3.5	23.1 ± 4.1	0.75
ART (*n*)	7 (20%)	1 (3%)	**< 0.05**
Cigarette smoking (*n*)	2 (6%)	3 (9%)	0.64
Previous CS (*n*)	11 (31%)	5 (14%)	**< 0.05**
Previous curettage (*n*)	8 (23%)	2 (6%)	**< 0.05**
Previous myomectomy (*n*)	3 (9%)	3 (9%)	-
Previous endometritis (*n*)	1 (3%)	0 (0%)	**< 0.0001**
GA delivery (weeks)	36.3 ± 1.4	38.9 ± 1.9	**< 0.0001**
Delivery by CS (*n*)	35 (100%)	6 (17%)	**< 0.0001**
Neonatal Weight (g)	2841 ± 464	3288 ± 458	**< 0.001**
Fetal sex			
*Male (n)*	18 (51%)	15 (43%)	0.47
*Female (n)*	17 (48%)	20 (57%)	0.47
Estimated Blood Loss (ml)	1311 ± 815	398 ± 320	**< 0.001**
Blood transfusion (*n*)	7 (20%)	0 (0%)	**< 0.0001**
Balloon (*n*)	9 (26%)	0 (0%)	**< 0.0001**
Hysterectomy (*n*)	4 (11%)	0 (0%)	**< 0.0001**
Placenta Accreta (*n*)	6 (17%)	0 (0%)	**< 0.0001**

Results are expressed as mean ± SD or percentage, according to variables, and were considered statistically significant for *p* < 0.05. *BMI*, body mass index; *ART*, assisted reproductive techniques; *CS*, caesarean section

### Plasma EVs from Normal Pregnant and PP Women Express PLAP

**Fig. 1:**
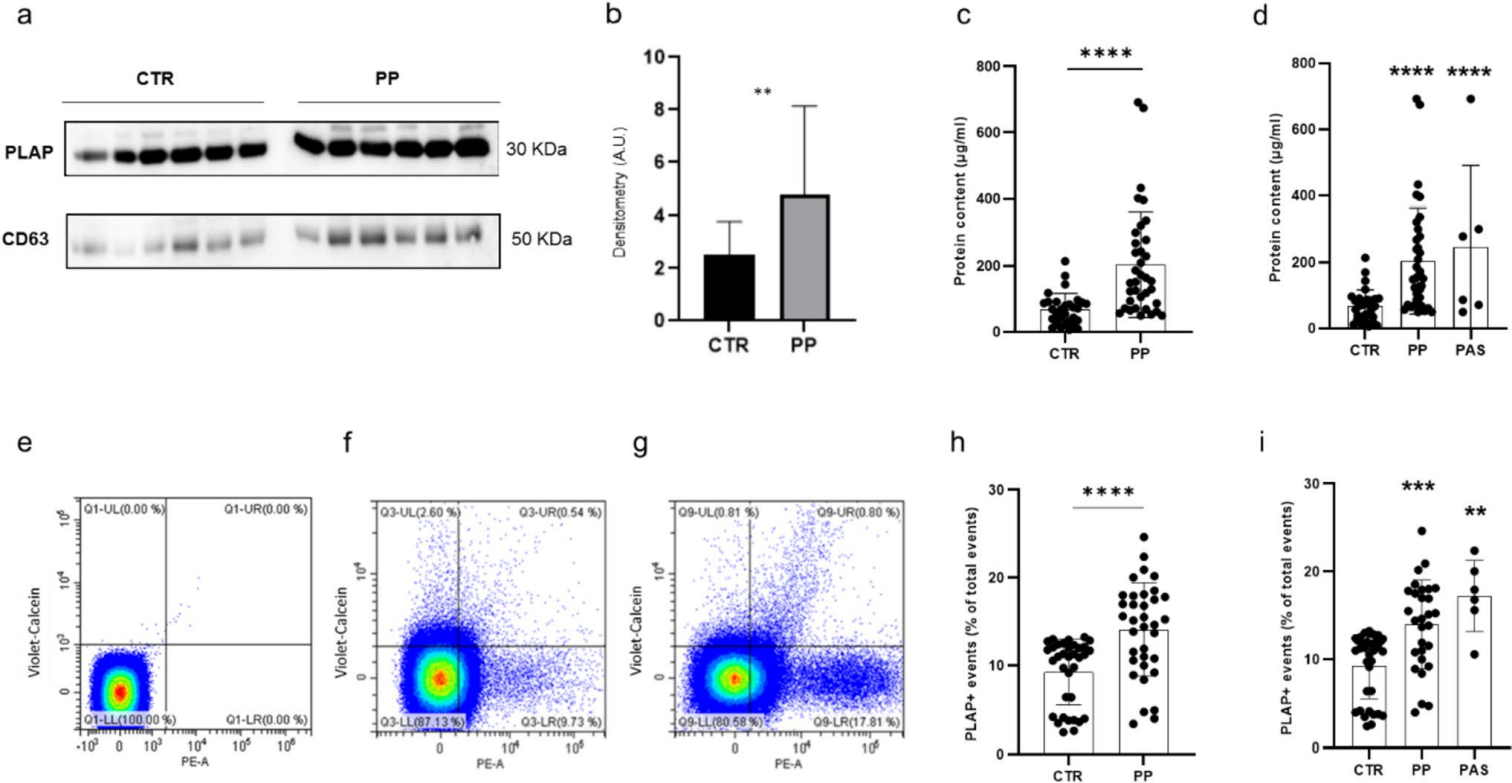
**a** Representative Western blot analysis for PLAP (30 kDa) and CD63 (50 KDa) expression of plasma EVs isolated from women with normal pregnancy (CTR; *n* = 6) and women with placenta previa (PP; *n* = 6), matched for gestational age. Protein content normalisation of samples was performed by ponceau. **b** Histogram showing densitometric analysis of PLAP protein expression in the Western blot showed in panel a. **c** and **d** Scatter graph representing the protein content of plasma EVs from CTR and PP. **c** Patients with PP (*n* = 35) showed a significantly higher plasma EVs protein content than CTR(*n* = 35). **d** Women with PP without PAS (*n* = 29) and PP and PAS (*n* = 6) showed significantly higher EVs protein content compared to CTR (*n* = 35). A trend of increased of EVs protein content in PP and PAS compared to PP without PAS was observed, although not reaching the statistical significance. **e**–**g** Representative experiment of cytofluorimetric analysis of STBEV obtained from negative control (**e**), a normal pregnant woman (**f**), and a woman diagnosed with PP (**g**), both at 35 weeks of gestation. The bottom right box shows the PLAP + EVs isolated from peripheral blood. **h** Box plots showing circulating levels of placenta-derived (PLAP +) EVs (STBEVs) isolated from plasma of CTR (*n* = 35) and PP (*n* = 35) cases. Patients with PP showed a significantly higher PLAP + content than CTR. **i** Sub-analysis of women with PP and PAS showed significantly higher levels of plasma STBEVs in both PP (*n* = 29) and PP and PAS (*n* = 6) cases compared to CTR (*n* = 35). STBEVs plasma levels in PP and PAS seemed to be increased compared to cases of PP without PAS, although not reaching the statistical significance. Results are expressed as mean ± SD. PLAP, placental alkaline phosphatase; CTR, controls; PP, placenta previa; A.U., arbitrary units. PAS, placenta accreta sectrum; EVs, extracellular vesicles; STBEVs, syncytiotrophoblast-derived EVs

The immunoblot performed on total EVs, isolated by ultracentrifugation from the plasma of both normal pregnant women and women with PP, matched for gestational age, demonstrated a frankly positive band for PLAP (30 KDa) and CD63 (50 KDa), therefore the presence of extracellular vesicles of placental origin in the plasma of both cases and controls (Fig. 1a).

### Protein Content of Plasma EVs from PP and PAS Women Is Higher than Controls

Bradford’s assay showed a significantly higher protein content in total EVs isolated from patients with PP compared to control patients (*p* < 0.05) (Fig. 1c), suggesting higher protein content cargo in STBEVs isolated from women with PP compared to controls. Sub-analysis of women with PP complicated by PAS compared to PP without PAS or controls showed an even higher plasma EVs protein content in cases of PAS compared to control, although not reaching a statistically significant difference compared to PP cases (Fig. 1d).

### Women with PP Show Higher Circulating Placenta-Derived EVs Compared to Controls by Flow Cytometry

In patients with PP, plasma circulating levels of placenta-derived vesicles (PLAP +) were significantly increased compared to control patients matched for gestational age (*p* < 0.001) (Fig. 1h). This difference was even higher when comparing the circulating STBEV levels of the subset of patients with PP and pathological diagnosis of PAS with controls (*p* < 0.001) (Fig. 1i).

## Discussion

In the present study, we aimed to assess circulating levels of STBEVs in a cohort of women with PP, diagnosed by ultrasound, with an expected high risk (11%) of PAS. Blood samples were collected at a gestational age between 28 and 37 weeks of gestation. We compared circulating levels of PP population with a cohort of normal pregnant women at the same gestational age. Here, we demonstrated that circulating levels of STBEVs are significantly higher in PP patients compared to controls. According to our hypothesis, we expected to find higher plasma levels of STBEVs in PAS cases compared to PP cases without PAS, because of a higher invasion of the decidua/myometrium and, thus, a more extended contact surface between maternal and placental circulation. Moreover, differences in terms of trophoblast histology in PAS disorders, like a significant higher presence of trophoblast inclusions, have been also suggested [16], supporting the hypothesis of a different placental architecture and feto-maternal interface in such a pathological condition. Unfortunately, we could only observe a trend of higher levels of plasma STBEVs in women with PP and PAS compared with PP cases, probably because of the small cohort of PP women analysed (35 cases) and the low incidence of PAS (6 cases) in our population. Recently, another research group has suggested a specific protein profile of circulating microparticles in PAS condition [17], although they did not focused specifically to placenta-derived vesicles.

This represents a pilot study, investigating a possible role of circulating STBEVs as a liquid biopsy of placental invasion abnormalities, ease to access in a not invasive fashion. Although PLAP protein can be expressed in some neoplastic conditions (gastric, pancreatic, endometrial, and ovarian cancer; https://www.proteinatlas.org/), it is a validated marker of placental derivation largely used for vesicles characterisation in pregnancy [12, 15]. Further larger multicentre studies (because of the low incidence of PAS) are required to assess longitudinal levels of STBEVs in the three trimesters of pregnancy in women at low and high risk of PP and PAS, later diagnosed with accreta by pathological examination. Furthermore, the analysis of the cargo of STBEVs in PAS could provide specific markers (proteins, miRNA) of abnormal placental invasion to be investigated from both physio-pathologic and diagnostic point of view.

We can speculate that in PP cases, without pathological diagnosis ex vivo of PAS, an abnormal adherence of the placenta can take place, because of the higher presence of decidual scars allowing abnormal adherence of the placenta to the maternal uterus, justifying, thus, the increased release of STBEVs into the maternal circulation found in vivo. In addition, in PP cases, higher syncytiotrophoblast stress can be expected, because of the unfavourable site of placental development at the low uterine segment.

## Conclusions

In conclusion, in this study, we observed, for the first time, that higher circulating levels of STBEV can be found in women with PP compared to those with normal placentation site at the same gestational age. Our data suggest a potential role for circulating STBEVs as a biological compartment to be investigated in the antenatal diagnosis of placental implantation abnormalities. Further studies are required to confirm our observation and to identify specific markers of PAS within the cargo of placental vesicles.

### Supplementary Information

Below is the link to the electronic supplementary material.

Supplementary file1 (PDF 24 KB)

## Data Availability

The data that support the findings of this study are available from the corresponding author, [CT], upon reasonable request.
