# Supplementary material for: Shedding of Syncytiotrophoblast-Derived Extracellular Vesicles Is Increased in Placenta Previa and Accreta Spectrum
Source: Reprod Sci. 2024 Mar 7;31(7):2043–8. doi: 10.1007/s43032-024-01491-1 (PMC11217103; doi:10.1007/s43032-024-01491-1)
Supplement: Supplementary file 1 — Supplementary file1 (PDF 24 KB) [file 43032_2024_1491_MOESM1_ESM.pdf]

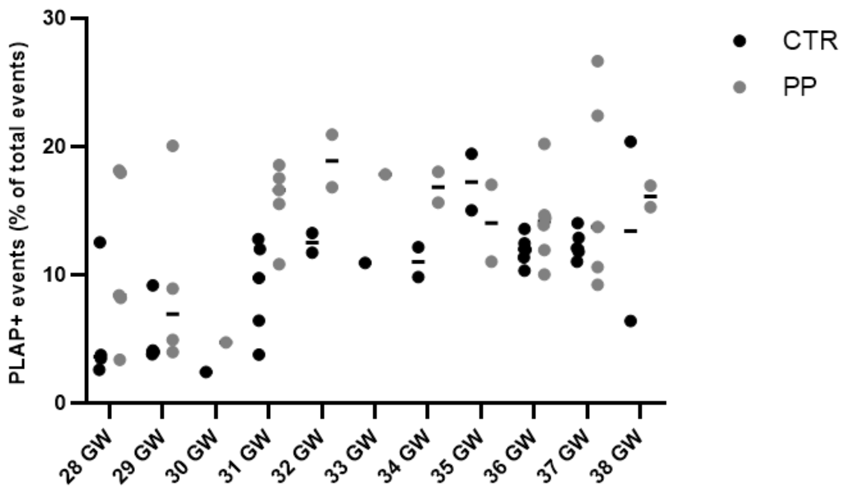

***Supplemental data**: Scatter graph showing circulating levels of placenta-derived (PLAP+) EVs (STBEVs) isolated from plasma of CTR (n=35) and PP (n=35) women analysed in relation to gestational week. Cases and controls were matched according to gestation. Results are expressed as mean. CTR: controls; PP: placenta previa; GW: gestational week.*
